# Linking chemical and disease entities to ontologies by integrating PageRank with extracted relations from literature

**DOI:** 10.1186/s13321-020-00461-4

**Published:** 2020-09-21

**Authors:** Pedro Ruas, Andre Lamurias, Francisco M. Couto

**Affiliations:** LASIGE, Faculdade de Ciências, Universidade de Lisboa, 1749-016 Lisbon, Portugal

**Keywords:** Named Entity Linking, Relation extraction, PageRank, Ontologies, Text mining

## Abstract

**Background:**

Named Entity Linking systems are a powerful aid to the manual curation of digital libraries, which is getting increasingly costly and inefficient due to the information overload. Models based on the Personalized PageRank (PPR) algorithm are one of the state-of-the-art approaches, but these have low performance when the disambiguation graphs are sparse.

**Findings:**

This work proposes a Named Entity Linking framework designated by Relation Extraction for Entity Linking (REEL) that uses automatically extracted relations to overcome this limitation. Our method builds a disambiguation graph, where the nodes are the ontology candidates for the entities and the edges are added according to the relations established in the text, which the method extracts automatically. The PPR algorithm and the information content of each ontology are then applied to choose the candidate for each entity that maximises the coherence of the disambiguation graph. We evaluated the method on three gold standards: the subset of the CRAFT corpus with ChEBI annotations (CRAFT-ChEBI), the subset of the BC5CDR corpus with disease annotations from the MEDIC vocabulary (BC5CDR-Diseases) and the subset with chemical annotations from the CTD-Chemical vocabulary (BC5CDR-Chemicals). The F1-Score achieved by REEL was 85.8%, 80.9% and 90.3% in these gold standards, respectively, outperforming baseline approaches.

**Conclusions:**

We demonstrated that RE tools can improve Named Entity Linking by capturing semantic information expressed in text missing in Knowledge Bases and use it to improve the disambiguation graph of Named Entity Linking models. REEL can be adapted to any text mining pipeline and potentially to any domain, as long as there is an ontology or other knowledge Base available.

## Introduction

### Background

There has been an intense growth in the amount of scientific literature available, mainly in the form of scientific articles, whose content is mostly expressed in natural language. For instance, there are more than 30 million articles in the PubMed repository [1], which is one of the most used libraries in the Life Sciences and the Biomedical domains. This information overload creates problems for researchers who want to retrieve information, because they need to spend more time and effort to find the relevant articles for their work. Simultaneously, the number of online resources of biological information has also been rising, as it is the case of the domain ontologies. Domain ontologies provide a coherent representation of the knowledge in a specific scientific field, allowing a standardised nomenclature to people from different backgrounds [2]. In order to keep these resources relevant, it is necessary to extract the information locked in scientific literature and transfer it to the ontologies, a highly complex task that is usually done by dedicated curators. With the increase of literature available, manual curation of these repositories gets more costly and inefficient. Text mining tools are thus essential to aid both researchers and curators in the extraction of relevant information from large amounts of text.

Named Entity Linking (NEL) is a typical text mining task (also designated by Named Entity Disambiguation or Normalisation), and its goal is to link each named entity in a given text to an appropriate identifier in a Knowledge Base (KB), i.e., to associate an entity mention with the KB concept that best represents it. NEL systems are a fundamental component of most text mining pipelines, usually used after the Named Entity Recognition (NER) systems and before the relation extraction (RE) systems. Some obstacles associated with NEL are the presence of entity name variations in the text, like abbreviations, acronyms, alternate spellings or synonyms, and the presence of entity ambiguity, since polysemous entity mentions can be linked to more than one KB concept according to their context [3]. For example, the entity mention “toxicity” can refer to “toxicity test”, a laboratory technique, or to “cardiac toxicity”, an adverse reaction. Additionally, NEL systems developed for scientific text have to deal with the high ambiguity arising from a lack of nomenclature standardisation and the high specificity of the language, which means that, in many cases, only an expert can understand the text content [4].

Many NEL approaches rely on the semantic information provided by the KBs, but in many cases the KBs lack important information. NEL is usually a preceding step of RE because it is useful to know the entities present in a given text before finding relations between them but the relations described in the text can also disclose semantic information that may not be expressed in the KBs. So, our hypothesis is that RE approaches can overcome the missing domain knowledge in the KBs and improve the performance of the NEL models that are highly dependant of the information provided by KBs.

We have previously developed the PPR-SSM model [5], a graph-based approach that applies the Personalized PageRank (PPR) algorithm and semantic similarity measures to perform the disambiguation of biomedical entity mentions to several ontologies. The model builds a disambiguation graph for each text, where the nodes are ontology candidates and the edges are based on the ontology structure. One of the main limitations we have detected is that, sometimes, the model creates incomplete or sparse disambiguation graphs, i.e., with too few edges between the nodes, which hampers the application of the PPR algorithm and impacts the overall precision of the model. As the edges in the disambiguation graph are added if the candidates are linked in the ontology, we can infer that the information provided by the ontology is not enough to build a dense graph.

The main contribution of the present work is a framework to improve the precision of graph-based NEL models, which we designate by Relationship Extraction for Entity Linking (REEL). This framework leverages the output of RE systems to build dense disambiguation graphs and to perform the disambiguation of disease and chemical entities. REEL was evaluated in several gold standards: the subset of the CRAFT corpus with Chemical Entities of Biological Interest (ChEBI) annotations (CRAFT-ChEBI) and the BC5CDR corpus with disease (MEDIC vocabulary) and chemical (CTD-Chemical vocabulary) annotations. The F1-Score obtained for the disambiguation of ChEBI, disease and chemical mentions was, respectively, 0.8577, 0.8086 and 0.9025. The comparison with two baseline approaches (a string matching technique and a modified version of PPR-SSM based solely on information provided by ontologies) shows that REEL can substantially improve the precision of graph-based NEL models.

### Related work

#### Local NEL models

The first NEL models relied on local approaches, i.e., assumed that each entity in a text should be disambiguated individually according to its lexical or semantic features. This approach is limited because many times the meaning of an entity varies according to the context where it appears. One example of this type of approach is Bunescu et al. [6], in which the authors explored the disambiguation of Wikipedia entities using Support Vector Machines (SVM).

#### Integrating global evidence in NEL models

More recent models assume that the entities in the same document must be somehow related, which means that the disambiguation of an entity influences the disambiguation of the others entities. PageRank is a Random Walks algorithm that was initially developed to measure the relative importance of web pages [7]. PageRank acts as a centrality measure in graphs or networks [8] and has been successfully adapted to the NEL task [9–11]. For example, Pershina et al. [11] proposed an approach based on the PPR algorithm that combines local and global features to assist in the disambiguation. For each document, this approach builds a disambiguation graph in which the nodes consist of Wikipedia candidates for the named entities and the edges are added according to the Wikipedia link structure. PPR is applied on the disambiguation graph and then ranks each node according to its contribution to the coherence of the graph. The model was evaluated on the dataset AIDA and achieved a disambiguation accuracy of 91.7%. Guo et al. [10] described a NEL method for Wikipedia entities that determines the local similarity between textual mentions and entities (using lexical and statistical features) and a disambiguation graph that maximises global coherence between the candidates for the entities in a document. The algorithm then performs Random Walks in the graph to derive the semantic similarity between every pair of entities. These two approaches share some similarities with our method, in the sense that both are graph-based models and both apply the Random Walks algorithm over a disambiguation graph to maximise the global coherence between entities in a given document. Besides, the models also include features to determine local similarity between each textual mentions and entities. Nevertheless, our method has noticeable differences to those approaches, namely, the edge generation in the disambiguation graph and the definition of the scoring function for the candidates.

Other NEL approaches consist in the application of different Deep Learning techniques, like Ganea et al. [12], which proposed a deep learning model that integrates local and global evidence to disambiguate entities at document level. The model includes entity embeddings to capture semantic information, a neural attention mechanism that selects words around the entity to help the disambiguation and a collective disambiguation module that uses a conditional random field for global inference in the document. Pre-trained language models, like BERT [13], create contextualised representations for entity mentions and have been fine-tuned for the NEL task [14, 15].

#### NEL models for biomedical text

There are fewer NEL models developed for biomedical text. Usually the community challenges are a good way to assess the state-of-the-art in the field. For instance, the BioCreativE (Critical Assessment of Information Extraction in Biology) challenge contains tasks related to biomedical digital curation, between them some related with NEL of biomedical entities, such as genes, chemicals and diseases. The description of the participating models in the latest edition can be consulted in Arighi et al. [16]. There are models that perform both named entity recognition and named entity linking of disease and chemical entities, such as TaggerOne [17] and DNorm [18]. D’Souza et al. [19] proposed an approach that performs the disambiguation of disease mentions and obtained an accuracy of 90.75 and 84.65 and in the ShARe/CLEF eHealth Challenge corpus and the NCBI Disease corpus. More recently, Ji et al. [20] fine-tuned the BERT model and two of its variants, ClinicalBert and BioBert, to the NEL task, evaluated them in the ShARe/CLEF, NCBI Disease and TAC2017ADR (drug labels) datasets and obtained an accuracy of 91.10%, 89.06% and 93.22%, respectively.

J-REED [21] is a model able to perform both NEL and RE. However, this approach performs both tasks sequentially and does not improve NEL with RE, which is the main goal of the present work. To the best of our knowledge, our method is the first attempt to use the RE output to improve the performance of graph-based NEL methods.

## Methods

### Definition of the NEL problem

**Fig. 1:**
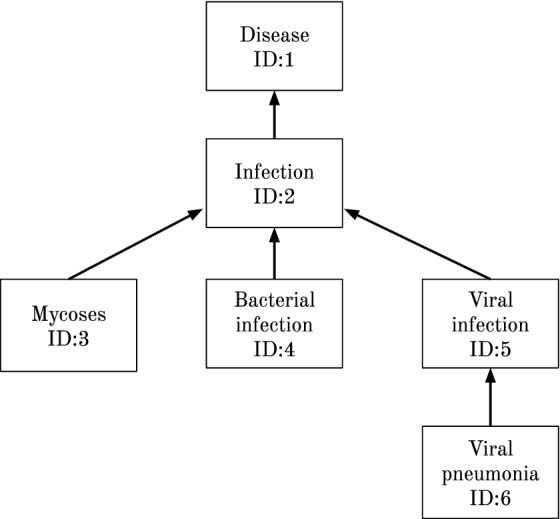
Subsumption relations. Example of a set of disease concepts and subsumption relations between them. The arrows denote the direction of the *is-a* relations

The starting requirement for NEL is a corpus containing documents with entity mentions already identified by a human annotator or by a NER system. The set of entity mentions in a given document is represented by *E*. The objective of NEL is to link each entity mention *e*, with $e \in E$, to the concept in a Knowledge Base (KB) that best represents it. The output of a NEL model consists of each entity mention associated with a KB identifier. A KB is a tuple $<C, R>$, where *C* is the set of concepts and *R* the set of relations between concepts. Each relation consists in a pair of concepts $(c_1, c_2)$, with $c_1,c_2 \in C$. An ontology is a type of KB that contains, among other types, subsumption or “is-a” relations between concepts (see Fig. 1).

The NEL task comprises two distinct steps:

- Candidate generation: Generation of the candidates list $CL(e)=\{c_e^{1},...,c_e^{i}| \forall c_e \in C\}$ for each entity mention *e* in set of document entity mentions *E*.
- Candidate ranking and disambiguation: Selection of the candidate ${c_e}$ in the candidate list *CL* that best represents each entity mention *e*, i.e., the highest ranked candidate.

### Candidate generation

#### Candidate list

The first step of NEL is accomplished through a search in the KB for each entity mentions (string matching technique). The candidates are ranked according to their lexical similarity which is determined by the edit distance, i.e, the minimum number of operations needed to convert one string into another. If there is an exact match between the entity mentions and any KB concept, the mentions is disambiguated with that concept and no candidates list is built. Otherwise, the first ten candidates are added to the candidates list. Additionally, if there are synonyms in the KB for the candidates, they are also added to the list. The baseline model in this work selects for each entity mentions the candidate with more lexical similarity. This approach is very limited, as it ignores the document context where the mentions appear: the candidate with the most similar string is not always the correct disambiguation and, consequently, a global approach will be more accurate. For that it is necessary to build a disambiguation graph.

#### KB-based disambiguation graph

The disambiguation graph *G* for a document is represented by $G = \{(e, c_e) | e \in E, c_e \in CL(e)\}$. Each node $(e, {c_e})$ in the graph is an entity mention/candidate pair and the edges between nodes are built according to the following link mode:

- KB-link: Two nodes $(e_1, {c_{e_1}})$ and $(e_2, {c_{e_2}})$ are connected in the graph if the candidates ${c_{e_1}}$ and ${c_{e_2}}$ are directly connected by the KB structure (the shortest path length between them is 1) and if ${e_1} \ne {e_2}$. This latter constraint is to prevent the generation of noisy edges between nodes, as only one node/candidate per entity mention constitutes the correct disambiguation. For example, “Viral pneumonia” and “Viral infection” in the example of the Fig. 1 are directly connected.

This method to build the disambiguation graph is the same used by our previous framework PPR-SSM [5] and other graph-based approaches [10, 11], in which the authors consider that an edge between two nodes or candidates occurs if the corresponding Wikipedia articles have at least one link between them.

The way the nodes are linked in the disambiguation graph directly affects the application of the PPR algorithm.

#### Improvement of the disambiguation graph with extracted relations

**Fig. 2:**
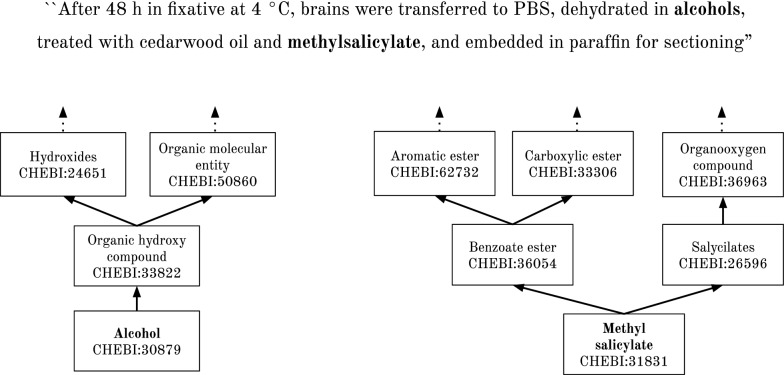
Relation between terms in the text. Example of a relation between the entity mentions “alcohols” and “methylsalicylate” that is described in an article, but it is not expressed in the ChEBI ontology structure. The closest ancestors of the respective ChEBI entities for these mentions, “Alcohol” and “Methyl salycilate”, and the respective ChEBI identifiers are also shown
- KB-Corpus-link: Two nodes $(e_1, {c_{e_1}})$ and $(e_2, {c_{e_2}})$ are connected if either appear in a relation described in text or if they are connected in the KB.

The lack of domain knowledge in the building of the disambiguation graphs is responsible for some limitations, such as the scarcity of edges between nodes. The PPR algorithm is a measure of centrality in the graph, so the calculation of the score for each node is directly related with the number of edges that traverse the nodes. Consequently, if the disambiguation graph has few edges between its nodes, the PPR algorithm will assign scores according to other criteria than the number of edges, like the number of descendants associated of the concept associated with the node or degree of the node, which does not properly assess the node contribution for the graph coherence. In many cases, the simple inclusion of KB relations between concepts is not enough to generate an adequate number of edges between nodes in the disambiguation graph. To overcome this problem, we propose to include information about entity relations described in a given corpus to generate the edges in the disambiguation graph. For that, two additional link modes between nodes in the disambiguation graph are defined:

- Corpus-link: Two nodes $(e_1, {c_{e_1}})$ and $(e_2, {c_{e_2}})$ are connected if the candidates ${c_{e_1}}$ and ${c_{e_2}}$ appear in a relation described in the text of the documents in the corpus and if ${e_1} \ne {e_2}$. In Fig. 2, the entity mentions “alcohols” and “methylsalicylate” are not linked in the structure of the ChEBI ontology, but there is an corpus document describing a relation between these two entities.

Besides the relations explicitly described in text that can be extracted by an RE tool, we also include in our approach human annotations of chemical-disease interactions whenever these are available in the corpus. In this case, we assume that there is a relation between any two given disease entities if the same chemical entity plays a role in both. Conversely, two chemical entities are related if they are involved with the same disease entity.

With the disambiguation graph already built, it is thus necessary to compute the weights for each node/candidate according to their relevance to the entities.

### Candidate ranking and disambiguation

The second step of NEL is the disambiguation of each entity mention *e* to a candidate ${c_e}$, which is determined by the function:

1 $$\begin{aligned} Disambiguate(e) = arg_{c_e} max\{score(e, c_e)\} \end{aligned}$$

The *score* function above determines the likelihood of the candidate ${c_e}$ being the correct disambiguation for entity mention *e*. The PageRank algorithm performs random walks in a graph and returns a probability distribution of reaching each node after a given number of iterations. In each iteration, there is a teleport probability $\epsilon$ of jumping to a random node in the graph, and a probability of 1 − $\epsilon$ (also called damping factor) of following an outgoing edge of the current node. In this way, the algorithm ranks each node, which can be considered a measure of centrality in the graph. When the teleports are not random but adjusted to the same source node, the PageRank algorithm is designated by Personalized PageRank (PPR). In the context of the NEL task, considering a given source node *s* and a given node *n* in the graph *G*, the PageRank score of the relation $PPR(s \rightarrow n)$ measures the relevance of node *n* for node *s*. The contribution of node *n* to the global coherence of the disambiguation graph *G* due to the presence of node *s* is expressed by the following equation:

2 $$\begin{aligned} Coherence_{s} (n) = PPR(s \rightarrow n) \end{aligned}$$

Thus, to determine the overall contribution of the node *n* to the global coherence it is necessary to sum all the contributions of the node related with the presence of the other source nodes (except the nodes representing candidates competing for the same entity mention as *n*):

3 $$\begin{aligned} Coherence(n) = \sum _{s \in G}coherence_{s}(n) \end{aligned}$$

The *coherence* expression in Eq. 3 constitutes the *score* function in Eq. 1. Intuitively, for each entity mention, the node/candidate that contributes the most for the global coherence of the disambiguation graph will be chosen to disambiguate the entity.

In order to add a layer of differentiation for the nodes in the disambiguation graph, the PageRank of each node *n* in relation to a source node *s* is multiplied by the information content (IC) of the node *n*:

4 $$\begin{aligned} Coherence_{s} (n) = PPR(s \rightarrow n) \cdot IC(n) \end{aligned}$$

The IC of a concept is a measure of its “rareness”: rare concepts will have higher information content. In the present work, we use the extrinsic IC definition, as described by Couto et Lamurias [22], in which the IC of a concept is associated with the frequency of its instances in an external dataset (for example a corpus). Pershina et al. [11] and Guo et al. [10] do not include the IC in their approach. Instead, Pershina et al. [11] use the Freebase popularity, which is a score based on the in-edges and the out-edges of pages in Wikipedia and Freebase.

### Models

To determine the impact of the relation extraction in the NEL performance, we evaluated the following models described below in several datasets:

- “String matching”: first baseline approach. For each entity to disambiguate, this model selects the candidate with highest lexical similarity (lowest edit distance) through string matching.
- “PPR-IC”: second baseline, corresponds roughly to PPR-SSM [5] without using semantic similarity measures. Consists in the application of the PPR algorithm with the inclusion of the IC for each node. The edges in the disambiguation graph are exclusively based on KB relations between concepts (link mode KB-link). The scoring function is Eq. 4.
- “REEL(Corpus)”: the application of the PPR-IC model, but using only relations described by the text to build the edge structure of the disambiguation graph (link mode Corpus-link). The scoring function is Eq. 4.
- “REEL(KB+Corpus)”: the application of the PPR-IC using both relations described by the text (link mode Corpus-link) or KB relations (link mode KB-link) to build the edge structure of the disambiguation graph. The scoring function is Eq. 4.

### Data description

The data used in this work consist of datasets/corpora and ontology files. The datasets contain the surface form of disease and chemical entities, and the respective ontology identifiers. The ontology files include information about the ontology concepts, as well the semantic relations between them.

#### Datasets

The “Colorado Richly Annotated Full-Text” (CRAFT) corpus is a set of 67 full-text biomedical articles from PubMed Central Open Access subset [23]. This gold standard contains, among others, 4548 manual annotations of ChEBI entities. The set of the corpus with ChEBI annotations will be further designed as “CRAFT-ChEBI”. In this work, we used the version 3.0 of this corpus [24].

To demonstrate that REEL can easily be adapted to include relations extracted by different tools, we evaluated the performance of the model on the BC5CDR corpus [25] openly available [26]. This gold standard was developed for the disease named entity recognition (DNER) task and the chemical-induced disease (CID) RE task in the for BioCreative V. This corpus consists in 1500 PubMed abstracts annotated with 4409 chemicals, 5818 diseases and 3116 chemical-disease interactions. The chemical annotations contain the respective MeSH unique ID from the “Chemicals and Drugs” category in the MeSH vocabulary, whereas the disease annotations contain the MeSH unique ID from “Diseases” category. The set of the corpus with disease annotations is further designated by “BC5CDR-Diseases” and the set of the corpus with chemical annotations by “BC5CDR-Chemicals”. We evaluated the models in the train, development and test sets and in a set containing all the corpus documents which we designate by “All”.

#### Ontologies

The first ontology we used was the ChEBI ontology, acronym for “Chemical Entities of Biological Interest”, which represents low-molecular weight chemical entities with biological relevance for living organisms [27]. As of 1 September, 2019 this repository contained 56090 annotated entries [28]. In the experiments described in this work we used the data from the release 179 [29].

The second ontology was the MEDIC Disease vocabulary from the Comparative Toxicogenomics Database (CTD), which is an hierarchical vocabulary that represents descriptors from the “Diseases” category of MeSH controlled vocabulary and genetic disorders from Online Mendelian Inheritance in Man (OMIM) repository [30]. As of May, 2020, this vocabulary contained 7246 entries representing distinct diseases entities [31] and in the experiments of this work we used the data from the referred month release [32].

The third ontology was the Chemicals vocabulary also from CTD, an hierarchical vocabulary representing descriptors from the “Chemicals and drugs” category of MESH. As of May, 2020, this vocabulary contained 16313 entries representing distinct chemical entities and in the experiments of this work we used the data from referred month release [33].

### Evaluation metrics

In each document of the corpus, repeated instances of an entity mention with the same surface form count as a unique entity. True positives (tp) refer to the number of entities correctly disambiguated, false positives (fp) to the number of entities wrongly disambiguated and false negatives (fn) to the number of entities that the model does not disambiguate. The performance of each model was evaluated in each dataset through the determination of the precision, recall and micro-averaged F1-score:

5 $$\begin{aligned}&Precision = \frac{tp}{tp + fp} \cdot 100 \end{aligned}$$

6 $$\begin{aligned}&Recall = \frac{tp}{tp + fn} \cdot 100 \end{aligned}$$

7 $$\begin{aligned}&F1-score = 2 \cdot \frac{precision \cdot recall}{precision + recall} \end{aligned}$$

### Implementation

#### Pre-processing

The module dedicated to the pre-processing of corpus documents was implemented in Python 3.6.8. This module generates the candidates lists through the FuzzyWuzzy Python library, i.e., for each entity mention the module obtains the first ten ontology concepts with more lexical similarity with the mention (and the synonyms for the candidates) and discards the candidates without a valid ontology identifier. The module also converts the ontologies into graph objects with the Networkx Python Library, which further allows the determination of the ontology relations between concepts. Alternatively, this module can also include information about relations between entities described in text, either from corpus annotations or from the output of any RE tool. In the end, the module creates a candidates file for each original document in the corpus, files that contain all the information necessary to build the disambiguation graph (nodes and edges).

#### BO-LSTM

To investigate the hypothesis that relations described in text can improve the edge generation in the disambiguation graph, we integrated the information obtained by BO-LSTM [34] in our NEL method. This RE tool applies a model based on a recurrent neural network with long short-term memory (LSTM) units to detect and classify relations between entities in text. In BO-LSTM multi-channel architecture the information used in the detection and classification of relations differs with the specific “channel” considered: shortest dependency path (SDP), WordNet classes or ChEBI ancestors. In the ChEBI ancestors channel, this model first links each entity mention to a ChEBI identifier through string matching and then builds a vector with the respective ChEBI ancestors. BO-LSTM was trained on the “SemEval 2013: Task 9 DDI extraction corpus” [35], that contains annotations of pharmacological substances and drug-drug interactions at the sentence level. It was later applied to the documents of CRAFT corpus using all the described channels in order to detect relations between every pair of ChEBI entities in a sentence. The output was a file with classification of each entity pair: “effect”, if there is an interaction or relation between the entities or “noeffect”, otherwise. For the link modes Corpus-link and KB-Corpus-link this information is in the candidates files and, consequently, in the edge structure of the respective disambiguation graph. For a more detailed description of BO-LSTM implementation, please refer to the original publication [34].

#### PPR

The input to this part of the model is the candidates files from pre-processing stage. The model uses the PPR implementation proposed by Pershina et al. [11]. The PPR algorithm was computed according to the Monte Carlo algorithm proposed by Fogaras et al. [36]. We decided to maintain the same values for the PPR parameters described by Pershina et al. [11]: initialisation with 2000 random walks for each source node, 5 steps of PPR and probability of jump to the source node (or teleport probability) of 0.2.

## Results and discussion

The evaluation results for the models in the different datasets are available in the Table 1.

**Table 1:** Evaluation results in the CRAFT-ChEBI dataset (top), BC5CDR-Diseases (middle) and BC5CDR-Chemicals (bottom)

Model	CRAFT-ChEBI											
	P	R	F1									
String matching	77.8	78.0	77.9									
PPR-IC	87.1	79.9	83.3									
REEL(Corpus)	*91.3*	*80.9*	*85.8*									
REEL(KB+Corpus)	*91.3*	*80.9*	*85.8*									

BC5CDR-Diseases												
Model	All			Train			Dev			Test		
	P	R	F1	P	R	F1	P	R	F1	P	R	F1
String matching	82.7	74.7	78.5	81.7	74.1	77.7	82.7	72.3	77.2	83.6	77.5	80.4
PPR-IC	83.4	74.9	78.9	84.1	74.6	79.1	86.2	73.0	79.1	87.2	78.2	82.5
REEL(Corpus)	*86.9*	*75.6*	*80.9*	87.8	75.4	81.1	87.9	*73.5*	*80.1*	*89.0*	*78.6*	*83.5*
REEL(KB+Corpus)	86.6	75.5	80.7	*88.2*	*75.5*	*81.4*	*88.0*	*73.5*	*80.1*	88.8	78.5	83.3

BC5CDR-Chemicals												
Model	All			Train			Dev			Test		
	P	R	F1	P	R	F1	P	R	F1	P	R	F1
String matching	94.9	84.1	89.2	93.7	84.4	88.8	95.3	85.2	90.0	95.3	82.6	88.5
PPR-IC	96.7	84.4	90.1	97.6	84.9	90.8	97.6	85.5	91.2	98.0	83.0	89.9
REEL(Corpus)	*97.0*	*84.4*	*90.3*	*98.0*	*85.0*	*91.0*	*98.3*	*85.6*	*91.5*	*98.4*	*83.0*	*90.0*
REEL(KB+Corpus)	*97.0*	*84.4*	*90.3*	*98.0*	*85.0*	*91.0*	98.2	*85.6*	*91.5*	*98.4*	*83.0*	*90.0*

For the datasets BC5CDR-Diseases and BC5CDR-Chemicals the results for each subset are shown. “All” refer to the entire corpus and “Train”, “Dev” and “Test” refer to the train, development and test sets, respectively

In the CRAFT-ChEBI dataset, the two REEL models achieved the same performance, a F1-Score of 85.8%, which is an improvement of 2.5% and 7.9% comparing with the “PPR-IC” and “String matching” baseline approaches. The precision achieved was 91.3%, an increase of 4.2% and 13.5% comparing with the “PPR-IC” and “String matching” models.

In the BC5CDR-Diseases dataset, the model “REEL(Corpus)” achieved the highest F1-Score, 80.9%, which represents an improvement of 2.0 % and 2.4% comparing with the “PPR-IC” and “String matching” models. The precision achieved was 86.9%, an increase of 3.5% and 4.2% comparing with the “PPR-IC” and “String matching” models. In the BC5CDR-Chemicals dataset, the models “REEL(Corpus)” and “REEL (KB+Corpus)” obtained the highest F1-Score, 90.3%, but the increase from the baseline approaches was lower: 0.2% and 1.1% comparing with the “PPR-IC” and “String matching” models. The precision increased by 0.3% and 2.1% from the precision of the “PPR-IC” and “String matching” models.

The two REEL models [“REEL(Corpus)” and “REEL(KB+Corpus)”] consistently achieved the best F1-Score in all datasets and respective sets. The higher F1-Score comparing with the baseline approaches is directly related with increases in the precision, as the recall did not substantially differ across models. These results demonstrate that the initial hypothesis of improving the precision of graph-based NEL models through RE is true.

The use of a RE tool (BO-LSTM) and the inclusion of chemical-disease interactions of the BC5CDR corpus overcame the lack of domain knowledge in the KB and originated denser disambiguation graphs, which by its turn, improved the performance of the PPR algorithm. The results obtained by REEL are explained by the fact that there is semantic information encoded in text and not expressed in the ontologies structure. For example, in the following sentence of document 14737183 of the CRAFT-ChEBI dataset “(...) skin from at/at mice reveal an abrupt dorsoventral transition of DOPA staining, which probably reflects the additive effects of reduced melanin content” there are two entities mentions: “DOPA”, annotated with the identifier “CHEBI:49168”, and “melanin”, annotated with the identifier “CHEBI:25179”. In the ChEBI structure these entities are not linked, but BO-LSTM can infer that the content of “melanin”’ affects the “DOPA staining” through contextual features.

**Fig. 3:**
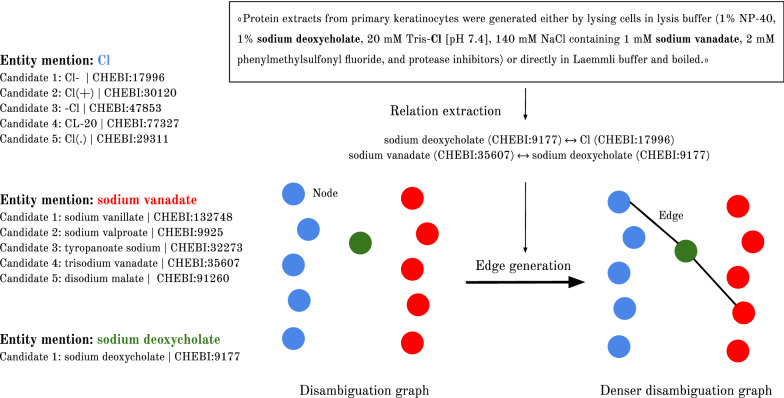
Disambiguation graph improved by extracted relations. Example showcasing the building of the disambiguation graph for three different entity mentions in a document of the CRAFT-ChEBI dataset and the further densification of the graph with extracted relations from the dataset

Another example, more complete, is shown in Fig. 3. In the document 15630473 of the CRAFT-ChEBI dataset there were, among others, the entity mentions “Cl”, “sodium vanate” and “sodium deoxycholate”. Only “sodium deoxycholate” had an exact match in ChEBI ontology, the homonymous concept with the identifier “CHEBI:9177”, the other two entity mentions had each one a candidate list with several ChEBI candidates (in the figure these candidate lists are abbreviated). Without the RE output and relying only in KB links, the disambiguation graph formed with the candidates for these entity mentions had no edges between the nodes. But BO-LSTM was able to extract relations between some of the candidates expressed elsewhere in the corpus and the addition of these relations to the disambiguation graph originated two new edges. The PPR algorithm assigns more weight to the nodes with higher degree (i.e. more interconnected) and in this case the nodes with higher degree (CHEBI:17996 and CHEBI:35607) corresponded to the correct disambiguation for the entity mentions.

The NEL performance of REEL is indirectly related with the performance of the RE tool that is used. The RE performance of BO-LSTM is lower than the inclusion of the gold standard annotations present in the BC5CDR corpus: BO-LSTM is not able to extract all the relations between entities present in the CRAFT-ChEBI dataset, contrarily to what happens in the BC5CDR corpus, where all the relations are known. The use of a RE tool is a more realistic scenario than the inclusion of gold standard annotations, because not always these are available, so we measured the NEL performance by REEL in these two different scenarios. According to Table 1, We can conclude that the NEL performance increases comparing with baseline approaches in these two different scenarios, so we can conclude that this increase in independent of the source of the extracted relations.

In the ChEBI ancestors channel of BO-LSTM, the model links each entity mention with a ChEBI identifier through string matching, which limits the performance of the tool when extracting the relations in text. So, the performance of REEL benefits with the BO-LSTM output, but could by its turn be used to improve BO-LSTM performance, more concretely by replacing the string matching method and improving its disambiguation component.

The REEL framework can be potentially adapted to any domain, as long as there is an ontology or other structured KB available. This feature is specially relevant for the biomedical and life sciences domains, where there is a lack of text mining tools, but there are many digital libraries with scientific information available. REEL could be used in any gold standard dataset as long it contains annotations of biomedical ontology concepts and in any biomedical text where the entities are already recognized by a Named Entity Recognition tool. The relations extracted in the CRAFT-ChEBI corpus (or in the BC5CDR corpus) could be included in REEL to improve its performance in that different gold standard/text. The framework only needs labelled data if it is necessary to train the RE tool, but if the tool is already trained or the relations are available, that need disappears.

### Error analysis

Despite the positive results achieved by REEL, there were some errors that prevented an even higher performance.

One type of error is associated with the presence of composite mentions in the BC5CDR-Diseases dataset. For example, the disease mention “detrimental effect on memory and cognition” is annotated with two different gold labels: “detrimental effect on memory” (D008569) and “detrimental effect on cognition” (D003072). REEL is not adapted to recognise and deal with this type of entity mentions because only one candidate is selected per entity.

The second type of error is related with the candidate generation step, as many entity mentions did not have the correct disambiguation in the respective candidates list, which impacted mainly the recall of the model. The string matching technique to generate candidates is useful to restrict the field of possible ontology candidates but sometimes leaves out correct candidates with little lexical overlap with the entity mention.

Another type of error is due to the presence of few entity mentions in a document. The PPR algorithm has higher performance in bigger and denser disambiguation graphs, but in certain cases, there is not enough entity mentions in a document to build a disambiguation graph with these characteristics.

## Conclusion

We developed REEL, which leverages extracted relations described in the text to build dense disambiguation graphs and then applies the PPR algorithm for candidate ranking and disambiguation. The framework was evaluated on three different gold standards, the CRAFT-ChEBI, the BC5CDR-Diseases and the BC5CDR-Chemicals datasets and achieved a F1-Score of 85.8%, 80.9% and 90.3%, respectively, which represents an improvement comparing with two baseline approaches. This improvement is due to increases in the precision.

The results show that REEL can be used to mitigate the problems associated with the application of PPR for NEL using sparse disambiguation graphs. Our framework improved the performance of NEL when the output of a deep-learning RE tool (BO-LSTM) is included and also when relations annotated in a gold standard (BC5CDR corpus) are included, which demonstrates that the framework have the flexibility to easily integrate relations provided by any source.

For future work, we intend to explore pre-trained language models, like BioBERT [37], to further improve the determination of local similarity between mentions and ontology concepts. Besides, it would be interesting to adapt this method to other types of KBs other than the ontologies, like Wikipedia, which are less formally defined.

## Data Availability

Project name: REEL. Project home page: https://github.com/lasigeBioTM/REEL. Operating system: Ubuntu 18.04 LTS. Programming language: Python $\ge$ 3.6, Bash. Other requirements: JDK $\ge$ 11.0.6, BO-LSTM. License: Apache License 2.0 The data supporting the results and the code necessary to reproduce the results are openly available on the referred GitHub repository, as well a Dockerfile to properly setup the environment to run the code. The data used in this work is available in the following links: ChEBI ontology file is available on ftp://ftp.ebi.ac.uk/pub/databases/chebi/archive/rel179/ontology/. CTD’s MEDIC Disease vocabulary and CTD’s Chemical Vocabulary files are available under conditions on http://www.ctdbase.org/reports/CTD_diseases.obo.gz and on http://www.ctdbase.org/reports/CTD_chemicals.tsv.gz, respectively. CRAFT Corpus is available on https://github.com/UCDenver-ccp/CRAFT/releases/download/3.0/craft-3.0.zip. BC5CDR corpus is available on https://github.com/JHnlp/BioCreative-V-CDR-Corpus/blob/master/CDR_Data.zip Alternatively, the script “get_data.sh” in the GitHub repository automatically downloads all the necessary data.

## Abbreviations

ChEBI: Chemical Elements of Biological Interest
CTD: Comparative Toxicogenomics Database
KB: Knowledge Base
IC: Information content
MeSH: Medical Subject Headings
NEL: Named Entity Linking
NER: Named Entity Recognition
PPR: Personalized PageRank
RE: Relation extraction
REEL: Relationship Extraction for Entity Linking
